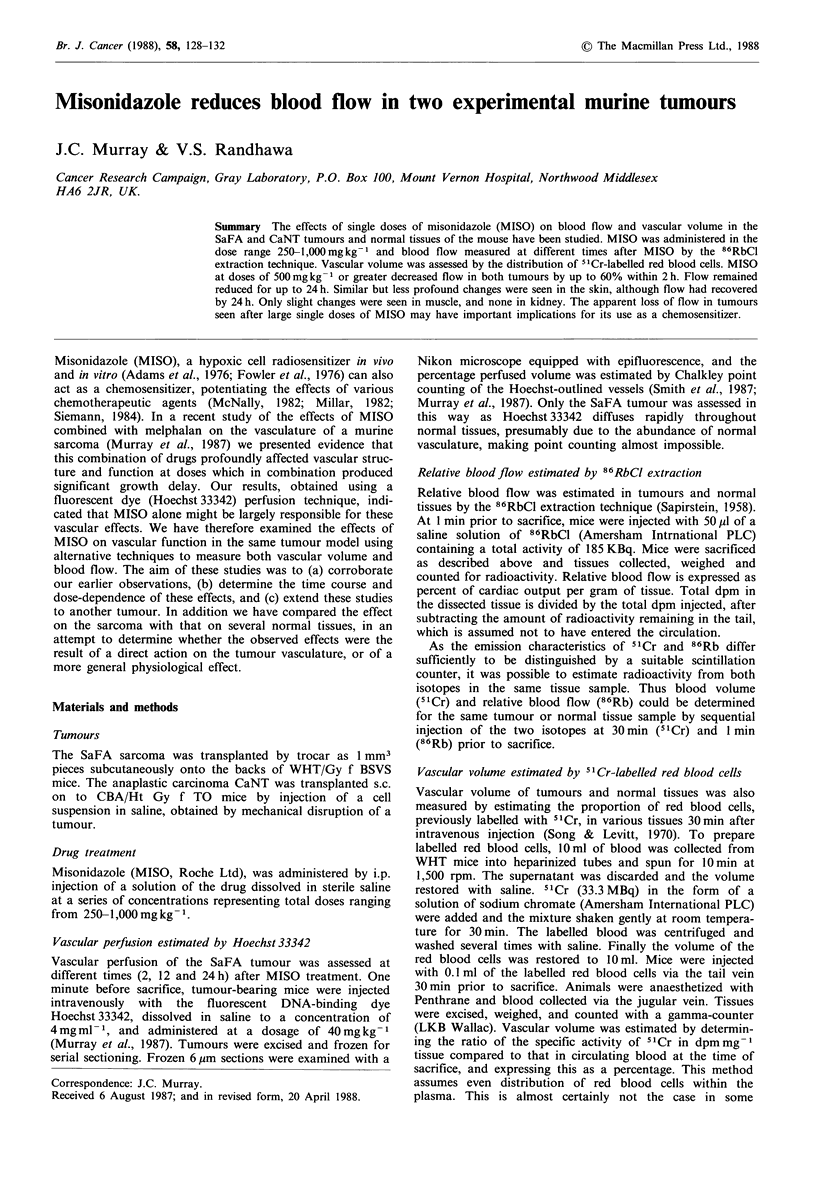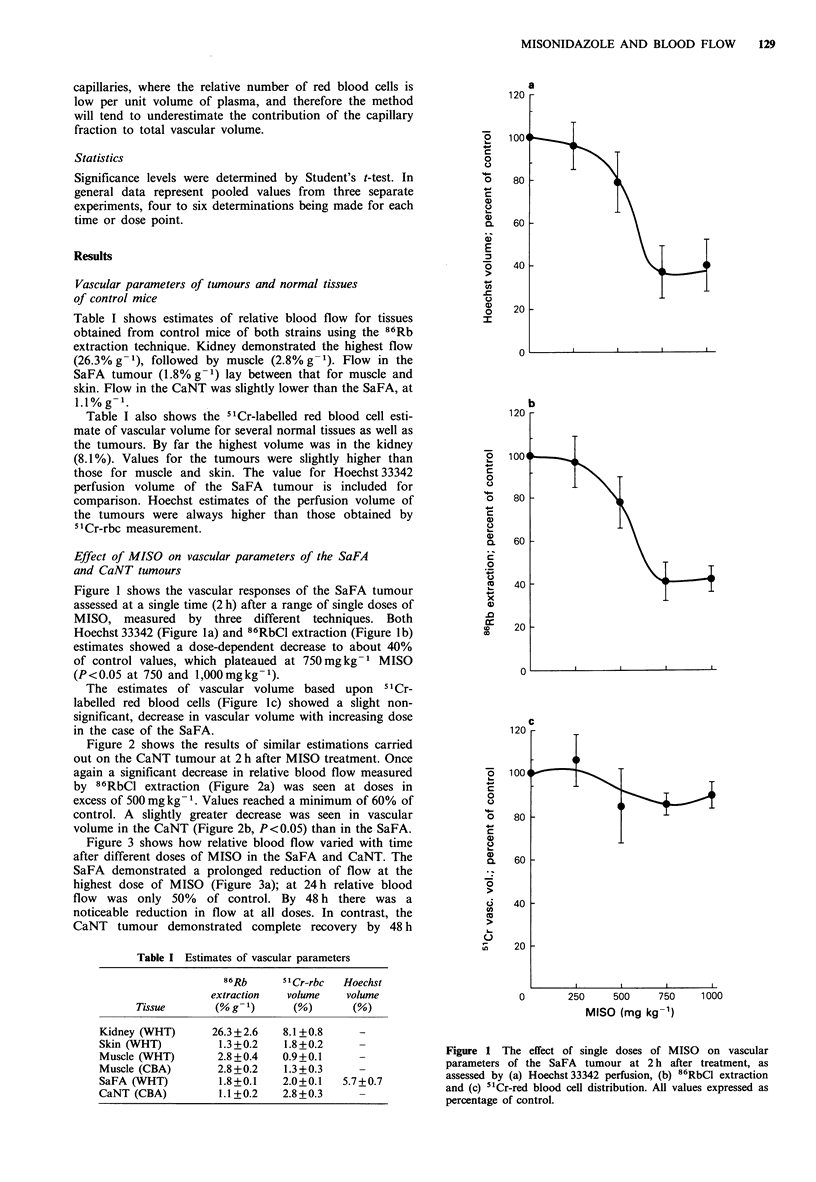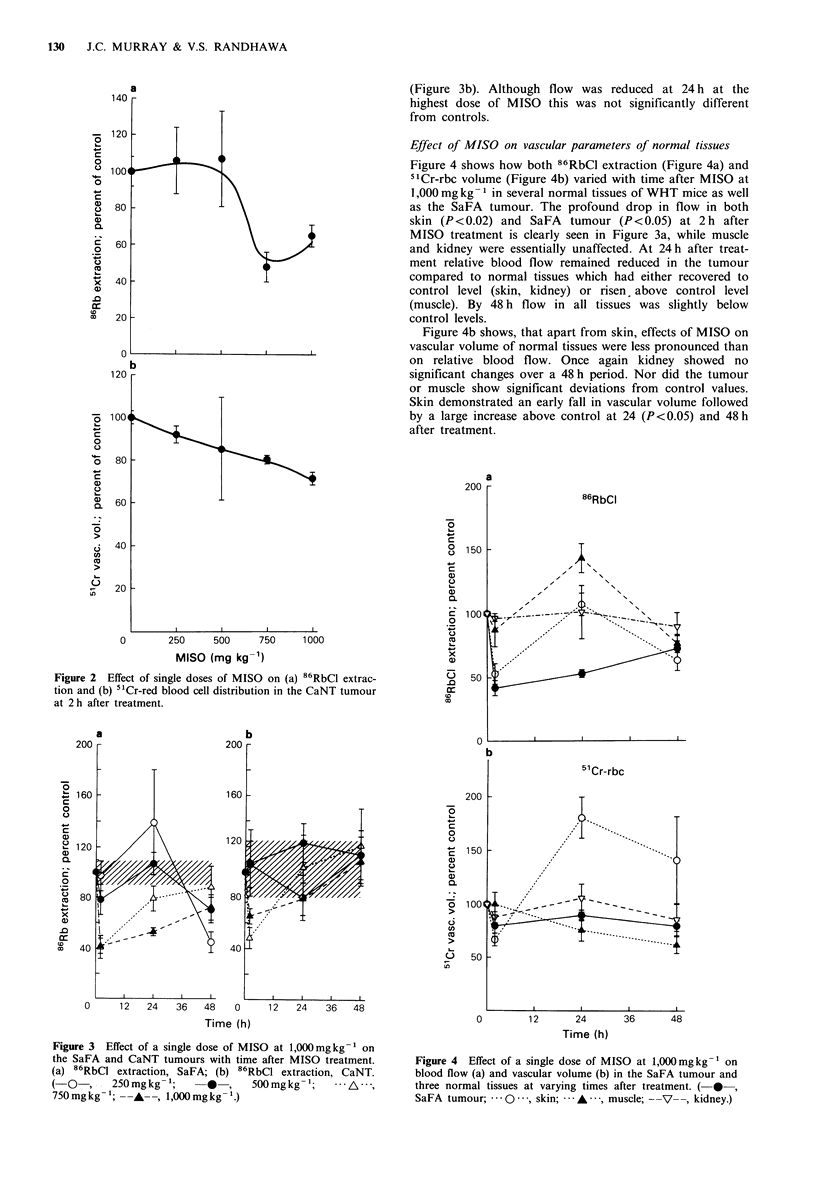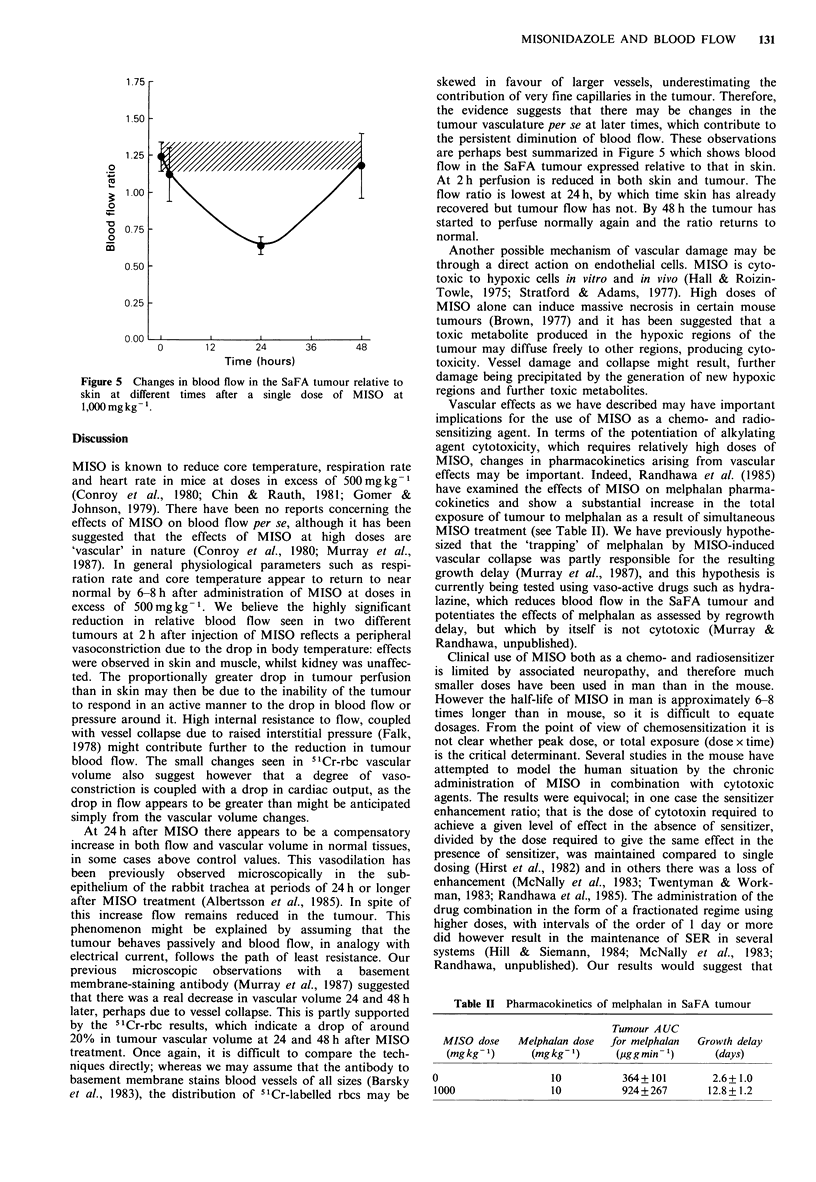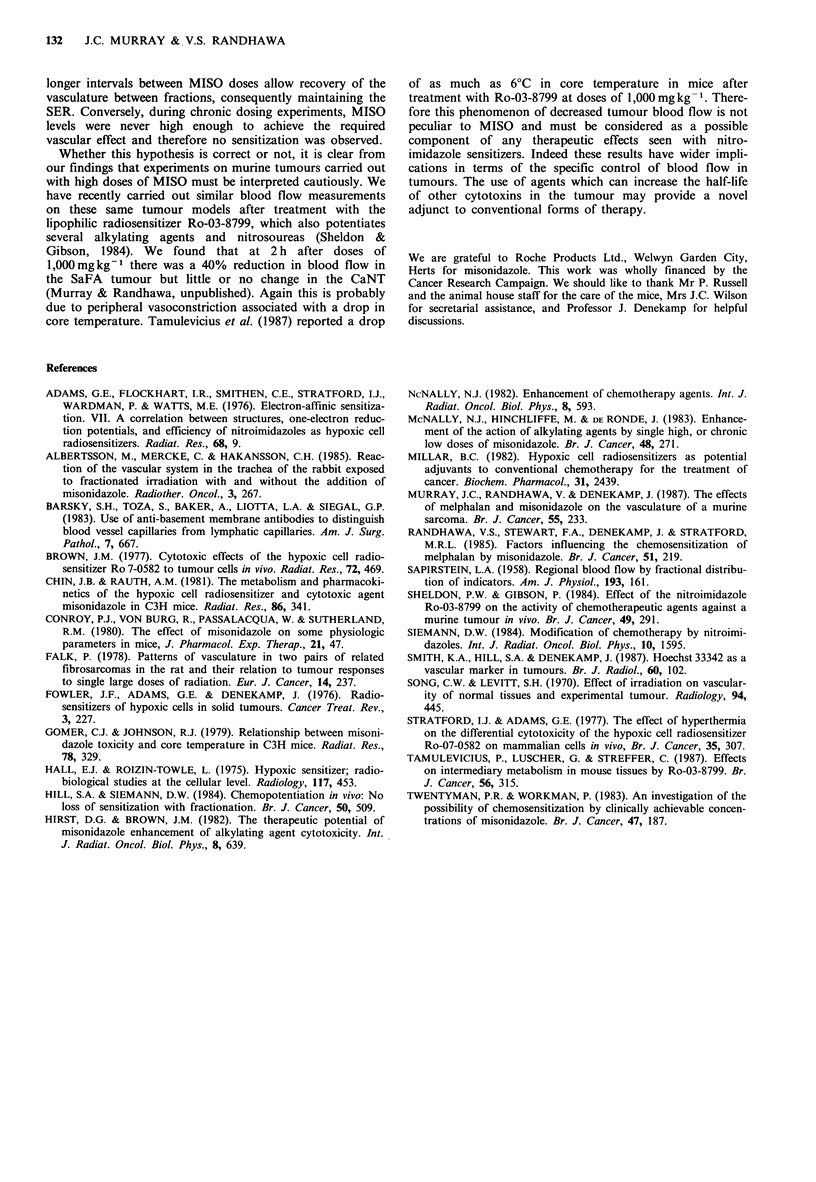# Misonidazole reduces blood flow in two experimental murine tumours.

**DOI:** 10.1038/bjc.1988.178

**Published:** 1988-08

**Authors:** J. C. Murray, V. S. Randhawa

**Affiliations:** Cancer Research Campaign, Gray Laboratory, Mount Vernon Hospital, Northwood Middlesex, UK.

## Abstract

The effects of single doses of misonidazole (MISO) on blood flow and vascular volume in the SaFA and CaNT tumours and normal tissues of the mouse have been studied. MISO was administered in the dose range 250-1,000 mg kg-1 and blood flow measured at different times after MISO by the 86RbCl extraction technique. Vascular volume was assessed by the distribution of 51Cr-labelled red blood cells. MISO at doses of 500 mg kg-1 or greater decreased flow in both tumours by up to 60% within 2 h. Flow remained reduced for up to 24 h. Similar but less profound changes were seen in the skin, although flow had recovered by 24 h. Only slight changes were seen in muscle, and none in kidney. The apparent loss of flow in tumours seen after large single doses of MISO may have important implications for its use as a chemosensitizer.


*Br. J. Cancer* (1988), **58**, 128–132 © The Macmillan Press Ltd., 1988

### Misonidazole reduces blood flow in two experimental murine tumours

J.C. Murray & V.S. Randhawa

*Cancer Research Campaign, Gray Laboratory, P.O. Box 100, Mount Vernon Hospital, Northwood Middlesex HA6 2JR, UK.*

**Summary** The effects of single doses of misonidazole (MISO) on blood flow and vascular volume in the SaFA and CaNT tumours and normal tissues of the mouse have been studied. MISO was administered in the dose range 250–1,000 mg kg$^{-1}$ and blood flow measured at different times after MISO by the $^{86}$RbCl extraction technique. Vascular volume was assessed by the distribution of $^{51}$Cr-labelled red blood cells. MISO at doses of 500 mg kg$^{-1}$ or greater decreased flow in both tumours by up to 60% within 2 h. Flow remained reduced for up to 24 h. Similar but less profound changes were seen in the skin, although flow had recovered by 24 h. Only slight changes were seen in muscle, and none in kidney. The apparent loss of flow in tumours seen after large single doses of MISO may have important implications for its use as a chemosensitizer.

Misonidazole (MISO), a hypoxic cell radiosensitizer *in vivo* and *in vitro* (Adams *et al.*, 1976; Fowler *et al.*, 1976) can also act as a chemosensitizer, potentiating the effects of various chemotherapeutic agents (McNally, 1982; Millar, 1982; Siemann, 1984). In a recent study of the effects of MISO combined with melphalan on the vasculature of a murine sarcoma (Murray *et al.*, 1987) we presented evidence that this combination of drugs profoundly affected vascular structure and function at doses which in combination produced significant growth delay. Our results, obtained using a fluorescent dye (Hoechst 33342) perfusion technique, indicated that MISO alone might be largely responsible for these vascular effects. We have therefore examined the effects of MISO on vascular function in the same tumour model using alternative techniques to measure both vascular volume and blood flow. The aim of these studies was to (a) corroborate our earlier observations, (b) determine the time course and dose-dependence of these effects, and (c) extend these studies to another tumour. In addition we have compared the effect on the sarcoma with that on several normal tissues, in an attempt to determine whether the observed effects were the result of a direct action on the tumour vasculature, or of a more general physiological effect.

### Materials and methods

### *Tumours*

The SaFA sarcoma was transplanted by trocar as 1 mm$^3$ pieces subcutaneously onto the backs of WHT/Gy f BSVS mice. The anaplastic carcinoma CaNT was transplanted s.c. on to CBA/Ht Gy f TO mice by injection of a cell suspension in saline, obtained by mechanical disruption of a tumour.

### *Drug treatment*

Misonidazole (MISO, Roche Ltd), was administered by i.p. injection of a solution of the drug dissolved in sterile saline at a series of concentrations representing total doses ranging from 250–1,000 mg kg$^{-1}$.

### *Vascular perfusion estimated by Hoechst 33342*

Vascular perfusion of the SaFA tumour was assessed at different times (2, 12 and 24 h) after MISO treatment. One minute before sacrifice, tumour-bearing mice were injected intravenously with the fluorescent DNA-binding dye Hoechst 33342, dissolved in saline to a concentration of 4 mg ml$^{-1}$, and administered at a dosage of 40 mg kg$^{-1}$ (Murray *et al.*, 1987). Tumours were excised and frozen for serial sectioning. Frozen 6 $\mu$m sections were examined with a Nikon microscope equipped with epifluorescence, and the percentage perfused volume was estimated by Chalkley point counting of the Hoechst-outlined vessels (Smith *et al.*, 1987; Murray *et al.*, 1987). Only the SaFA tumour was assessed in this way as Hoechst 33342 diffuses rapidly throughout normal tissues, presumably due to the abundance of normal vasculature, making point counting almost impossible.

### *Relative blood flow estimated by $^{86}$RbCl extraction*

Relative blood flow was estimated in tumours and normal tissues by the $^{86}$RbCl extraction technique (Sapirstein, 1958). At 1 min prior to sacrifice, mice were injected with 50 $\mu$l of a saline solution of $^{86}$RbCl (Amersham Intrnational PLC) containing a total activity of 185 KBq. Mice were sacrificed as described above and tissues collected, weighed and counted for radioactivity. Relative blood flow is expressed as percent of cardiac output per gram of tissue. Total dpm in the dissected tissue is divided by the total dpm injected, after subtracting the amount of radioactivity remaining in the tail, which is assumed not to have entered the circulation.

As the emission characteristics of $^{51}$Cr and $^{86}$Rb differ sufficiently to be distinguished by a suitable scintillation counter, it was possible to estimate radioactivity from both isotopes in the same tissue sample. Thus blood volume ($^{51}$Cr) and relative blood flow ($^{86}$Rb) could be determined for the same tumour or normal tissue sample by sequential injection of the two isotopes at 30 min ($^{51}$Cr) and 1 min ($^{86}$Rb) prior to sacrifice.

### *Vascular volume estimated by $^{51}$Cr-labelled red blood cells*

Vascular volume of tumours and normal tissues was also measured by estimating the proportion of red blood cells, previously labelled with $^{51}$Cr, in various tissues 30 min after intravenous injection (Song & Levitt, 1970). To prepare labelled red blood cells, 10 ml of blood was collected from WHT mice into heparinized tubes and spun for 10 min at 1,500 rpm. The supernatant was discarded and the volume restored with saline. $^{51}$Cr (33.3 MBq) in the form of a solution of sodium chromate (Amersham International PLC) were added and the mixture shaken gently at room temperature for 30 min. The labelled blood was centrifuged and washed several times with saline. Finally the volume of the red blood cells was restored to 10 ml. Mice were injected with 0.1 ml of the labelled red blood cells via the tail vein 30 min prior to sacrifice. Animals were anaesthetized with Penthrane and blood collected via the jugular vein. Tissues were excised, weighed, and counted with a gamma-counter (LKB Wallac). Vascular volume was estimated by determining the ratio of the specific activity of $^{51}$Cr in dpm mg$^{-1}$ tissue compared to that in circulating blood at the time of sacrifice, and expressing this as a percentage. This method assumes even distribution of red blood cells within the plasma. This is almost certainly not the case in some

Correspondence: J.C. Murray.
Received 6 August 1987; and in revised form, 20 April 1988.

capillaries, where the relative number of red blood cells is low per unit volume of plasma, and therefore the method will tend to underestimate the contribution of the capillary fraction to total vascular volume.

*Statistics*

Significance levels were determined by Student's *t*-test. In general data represent pooled values from three separate experiments, four to six determinations being made for each time or dose point.

### Results

*Vascular parameters of tumours and normal tissues of control mice*

Table I shows estimates of relative blood flow for tissues obtained from control mice of both strains using the $^{86}$Rb extraction technique. Kidney demonstrated the highest flow (26.3% $g^{-1}$), followed by muscle (2.8% $g^{-1}$). Flow in the SaFA tumour (1.8% $g^{-1}$) lay between that for muscle and skin. Flow in the CaNT was slightly lower than the SaFA, at 1.1% $g^{-1}$.

Table I also shows the $^{51}$Cr-labelled red blood cell estimate of vascular volume for several normal tissues as well as the tumours. By far the highest volume was in the kidney (8.1%). Values for the tumours were slightly higher than those for muscle and skin. The value for Hoechst 33342 perfusion volume of the SaFA tumour is included for comparison. Hoechst estimates of the perfusion volume of the tumours were always higher than those obtained by $^{51}$Cr-rbc measurement.

*Effect of MISO on vascular parameters of the SaFA and CaNT tumours*

Figure 1 shows the vascular responses of the SaFA tumour assessed at a single time (2 h) after a range of single doses of MISO, measured by three different techniques. Both Hoechst 33342 (Figure 1a) and $^{86}$RbCl extraction (Figure 1b) estimates showed a dose-dependent decrease to about 40% of control values, which plateaued at 750 mg $kg^{-1}$ MISO ($P<0.05$ at 750 and 1,000 mg $kg^{-1}$).

The estimates of vascular volume based upon $^{51}$Cr-labelled red blood cells (Figure 1c) showed a slight non-significant, decrease in vascular volume with increasing dose in the case of the SaFA.

Figure 2 shows the results of similar estimations carried out on the CaNT tumour at 2 h after MISO treatment. Once again a significant decrease in relative blood flow measured by $^{86}$RbCl extraction (Figure 2a) was seen at doses in excess of 500 mg $kg^{-1}$. Values reached a minimum of 60% of control. A slightly greater decrease was seen in vascular volume in the CaNT (Figure 2b, $P<0.05$) than in the SaFA.

Figure 3 shows how relative blood flow varied with time after different doses of MISO in the SaFA and CaNT. The SaFA demonstrated a prolonged reduction of flow at the highest dose of MISO (Figure 3a); at 24 h relative blood flow was only 50% of control. By 48 h there was a noticeable reduction in flow at all doses. In contrast, the CaNT tumour demonstrated complete recovery by 48 h

**Table I** Estimates of vascular parameters

| *Tissue* | *$^{86}$Rb extraction ($\% g^{-1}$)* | *$^{51}$Cr-rbc volume (%)* | *Hoechst volume (%)* |
|---|---|---|---|
| Kidney (WHT) | 26.3±2.6 | 8.1±0.8 | – |
| Skin (WHT) | 1.3±0.2 | 1.8±0.2 | – |
| Muscle (WHT) | 2.8±0.4 | 0.9±0.1 | – |
| Muscle (CBA) | 2.8±0.2 | 1.3±0.3 | – |
| SaFA (WHT) | 1.8±0.1 | 2.0±0.1 | 5.7±0.7 |
| CaNT (CBA) | 1.1±0.2 | 2.8±0.3 | – |

**Figure 1** The effect of single doses of MISO on vascular parameters of the SaFA tumour at 2 h after treatment, as assessed by (a) Hoechst 33342 perfusion, (b) $^{86}$RbCl extraction and (c) $^{51}$Cr-red blood cell distribution. All values expressed as percentage of control.

**Figure 2** Effect of single doses of MISO on (a) $^{86}$RbCl extraction and (b) $^{51}$Cr-red blood cell distribution in the CaNT tumour at 2 h after treatment.

**Figure 3** Effect of a single dose of MISO at 1,000 mg kg$^{-1}$ on the SaFA and CaNT tumours with time after MISO treatment. (a) $^{86}$RbCl extraction, SaFA; (b) $^{86}$RbCl extraction, CaNT. (—○—, 250 mg kg$^{-1}$; —●—, 500 mg kg$^{-1}$; ···△···, 750 mg kg$^{-1}$; --▲--, 1,000 mg kg$^{-1}$.)

(Figure 3b). Although flow was reduced at 24 h at the highest dose of MISO this was not significantly different from controls.

*Effect of MISO on vascular parameters of normal tissues*

Figure 4 shows how both $^{86}$RbCl extraction (Figure 4a) and $^{51}$Cr-rbc volume (Figure 4b) varied with time after MISO at 1,000 mg kg$^{-1}$ in several normal tissues of WHT mice as well as the SaFA tumour. The profound drop in flow in both skin ($P<0.02$) and SaFA tumour ($P<0.05$) at 2 h after MISO treatment is clearly seen in Figure 3a, while muscle and kidney were essentially unaffected. At 24 h after treatment relative blood flow remained reduced in the tumour compared to normal tissues which had either recovered to control level (skin, kidney) or risen, above control level (muscle). By 48 h flow in all tissues was slightly below control levels.

Figure 4b shows, that apart from skin, effects of MISO on vascular volume of normal tissues were less pronounced than on relative blood flow. Once again kidney showed no significant changes over a 48 h period. Nor did the tumour or muscle show significant deviations from control values. Skin demonstrated an early fall in vascular volume followed by a large increase above control at 24 ($P<0.05$) and 48 h after treatment.

**Figure 4** Effect of a single dose of MISO at 1,000 mg kg$^{-1}$ on blood flow (a) and vascular volume (b) in the SaFA tumour and three normal tissues at varying times after treatment. (—●—, SaFA tumour; ···○···, skin; ···▲···, muscle; --▽--, kidney.)

**Figure 5** Changes in blood flow in the SaFA tumour relative to skin at different times after a single dose of MISO at 1,000 mg kg$^{-1}$.

### Discussion

MISO is known to reduce core temperature, respiration rate and heart rate in mice at doses in excess of 500 mg kg$^{-1}$ (Conroy *et al.*, 1980; Chin & Rauth, 1981; Gomer & Johnson, 1979). There have been no reports concerning the effects of MISO on blood flow *per se*, although it has been suggested that the effects of MISO at high doses are 'vascular' in nature (Conroy *et al.*, 1980; Murray *et al.*, 1987). In general physiological parameters such as respiration rate and core temperature appear to return to near normal by 6–8 h after administration of MISO at doses in excess of 500 mg kg$^{-1}$. We believe the highly significant reduction in relative blood flow seen in two different tumours at 2 h after injection of MISO reflects a peripheral vasoconstriction due to the drop in body temperature: effects were observed in skin and muscle, whilst kidney was unaffected. The proportionally greater drop in tumour perfusion than in skin may then be due to the inability of the tumour to respond in an active manner to the drop in blood flow or pressure around it. High internal resistance to flow, coupled with vessel collapse due to raised interstitial pressure (Falk, 1978) might contribute further to the reduction in tumour blood flow. The small changes seen in $^{51}$Cr-rbc vascular volume also suggest however that a degree of vasoconstriction is coupled with a drop in cardiac output, as the drop in flow appears to be greater than might be anticipated simply from the vascular volume changes.

At 24 h after MISO there appears to be a compensatory increase in both flow and vascular volume in normal tissues, in some cases above control values. This vasodilation has been previously observed microscopically in the subepithelium of the rabbit trachea at periods of 24 h or longer after MISO treatment (Albertsson *et al.*, 1985). In spite of this increase flow remains reduced in the tumour. This phenomenon might be explained by assuming that the tumour behaves passively and blood flow, in analogy with electrical current, follows the path of least resistance. Our previous microscopic observations with a basement membrane-staining antibody (Murray *et al.*, 1987) suggested that there was a real decrease in vascular volume 24 and 48 h later, perhaps due to vessel collapse. This is partly supported by the $^{51}$Cr-rbc results, which indicate a drop of around 20% in tumour vascular volume at 24 and 48 h after MISO treatment. Once again, it is difficult to compare the techniques directly; whereas we may assume that the antibody to basement membrane stains blood vessels of all sizes (Barsky *et al.*, 1983), the distribution of $^{51}$Cr-labelled rbcs may be skewed in favour of larger vessels, underestimating the contribution of very fine capillaries in the tumour. Therefore, the evidence suggests that there may be changes in the tumour vasculature *per se* at later times, which contribute to the persistent diminution of blood flow. These observations are perhaps best summarized in Figure 5 which shows blood flow in the SaFA tumour expressed relative to that in skin. At 2 h perfusion is reduced in both skin and tumour. The flow ratio is lowest at 24 h, by which time skin has already recovered but tumour flow has not. By 48 h the tumour has started to perfuse normally again and the ratio returns to normal.

Another possible mechanism of vascular damage may be through a direct action on endothelial cells. MISO is cytotoxic to hypoxic cells *in vitro* and *in vivo* (Hall & Roizin-Towle, 1975; Stratford & Adams, 1977). High doses of MISO alone can induce massive necrosis in certain mouse tumours (Brown, 1977) and it has been suggested that a toxic metabolite produced in the hypoxic regions of the tumour may diffuse freely to other regions, producing cytotoxicity. Vessel damage and collapse might result, further damage being precipitated by the generation of new hypoxic regions and further toxic metabolites.

Vascular effects as we have described may have important implications for the use of MISO as a chemo- and radiosensitizing agent. In terms of the potentiation of alkylating agent cytotoxicity, which requires relatively high doses of MISO, changes in pharmacokinetics arising from vascular effects may be important. Indeed, Randhawa *et al.* (1985) have examined the effects of MISO on melphalan pharmacokinetics and show a substantial increase in the total exposure of tumour to melphalan as a result of simultaneous MISO treatment (see Table II). We have previously hypothesized that the 'trapping' of melphalan by MISO-induced vascular collapse was partly responsible for the resulting growth delay (Murray *et al.*, 1987), and this hypothesis is currently being tested using vaso-active drugs such as hydralazine, which reduces blood flow in the SaFA tumour and potentiates the effects of melphalan as assessed by regrowth delay, but which by itself is not cytotoxic (Murray & Randhawa, unpublished).

Clinical use of MISO both as a chemo- and radiosensitizer is limited by associated neuropathy, and therefore much smaller doses have been used in man than in the mouse. However the half-life of MISO in man is approximately 6–8 times longer than in mouse, so it is difficult to equate dosages. From the point of view of chemosensitization it is not clear whether peak dose, or total exposure (dose x time) is the critical determinant. Several studies in the mouse have attempted to model the human situation by the chronic administration of MISO in combination with cytotoxic agents. The results were equivocal; in one case the sensitizer enhancement ratio; that is the dose of cytotoxin required to achieve a given level of effect in the absence of sensitizer, divided by the dose required to give the same effect in the presence of sensitizer, was maintained compared to single dosing (Hirst *et al.*, 1982) and in others there was a loss of enhancement (McNally *et al.*, 1983; Twentyman & Workman, 1983; Randhawa *et al.*, 1985). The administration of the drug combination in the form of a fractionated regime using higher doses, with intervals of the order of 1 day or more did however result in the maintenance of SER in several systems (Hill & Siemann, 1984; McNally *et al.*, 1983; Randhawa, unpublished). Our results would suggest that

**Table II** Pharmacokinetics of melphalan in SaFA tumour

| *MISO dose (mg kg$^{-1}$)* | *Melphalan dose (mg kg$^{-1}$)* | *Tumour AUC for melphalan (μg g min$^{-1}$)* | *Growth delay (days)* |
|---|---|---|---|
| 0 | 10 | 364±101 | 2.6±1.0 |
| 1000 | 10 | 924±267 | 12.8±1.2 |

longer intervals between MISO doses allow recovery of the vasculature between fractions, consequently maintaining the SER. Conversely, during chronic dosing experiments, MISO levels were never high enough to achieve the required vascular effect and therefore no sensitization was observed.

Whether this hypothesis is correct or not, it is clear from our findings that experiments on murine tumours carried out with high doses of MISO must be interpreted cautiously. We have recently carried out similar blood flow measurements on these same tumour models after treatment with the lipophilic radiosensitizer Ro-03-8799, which also potentiates several alkylating agents and nitrosoureas (Sheldon & Gibson, 1984). We found that at 2 h after doses of 1,000 mg $kg^{-1}$ there was a 40% reduction in blood flow in the SaFA tumour but little or no change in the CaNT (Murray & Randhawa, unpublished). Again this is probably due to peripheral vasoconstriction associated with a drop in core temperature. Tamulevicius *et al.* (1987) reported a drop of as much as 6°C in core temperature in mice after treatment with Ro-03-8799 at doses of 1,000 mg $kg^{-1}$. Therefore this phenomenon of decreased tumour blood flow is not peculiar to MISO and must be considered as a possible component of any therapeutic effects seen with nitroimidazole sensitizers. Indeed these results have wider implications in terms of the specific control of blood flow in tumours. The use of agents which can increase the half-life of other cytotoxins in the tumour may provide a novel adjunct to conventional forms of therapy.

We are grateful to Roche Products Ltd., Welwyn Garden City, Herts for misonidazole. This work was wholly financed by the Cancer Research Campaign. We should like to thank Mr P. Russell and the animal house staff for the care of the mice, Mrs J.C. Wilson for secretarial assistance, and Professor J. Denekamp for helpful discussions.

### References

ADAMS, G.E., FLOCKHART, I.R., SMITHEN, C.E., STRATFORD, I.J., WARDMAN, P. & WATTS, M.E. (1976). Electron-affinic sensitization. VII. A correlation between structures, one-electron reduction potentials, and efficiency of nitroimidazoles as hypoxic cell radiosensitizers. *Radiat. Res.*, **68**, 9.

ALBERTSSON, M., MERCKE, C. & HAKANSSON, C.H. (1985). Reaction of the vascular system in the trachea of the rabbit exposed to fractionated irradiation with and without the addition of misonidazole. *Radiother. Oncol.*, **3**, 267.

BARSKY, S.H., TOZA, S., BAKER, A., LIOTTA, L.A. & SIEGAL, G.P. (1983). Use of anti-basement membrane antibodies to distinguish blood vessel capillaries from lymphatic capillaries. *Am. J. Surg. Pathol.*, **7**, 667.

BROWN, J.M. (1977). Cytotoxic effects of the hypoxic cell radiosensitizer Ro 7-0582 to tumour cells *in vivo*. *Radiat. Res.*, **72**, 469.

CHIN, J.B. & RAUTH, A.M. (1981). The metabolism and pharmacokinetics of the hypoxic cell radiosensitizer and cytotoxic agent misonidazole in C3H mice. *Radiat. Res.*, **86**, 341.

CONROY, P.J., VON BURG, R., PASSALACQUA, W. & SUTHERLAND, R.M. (1980). The effect of misonidazole on some physiologic parameters in mice, *J. Pharmacol. Exp. Therap.*, **21**, 47.

FALK, P. (1978). Patterns of vasculature in two pairs of related fibrosarcomas in the rat and their relation to tumour responses to single large doses of radiation. *Eur. J. Cancer*, **14**, 237.

FOWLER, J.F., ADAMS, G.E. & DENEKAMP, J. (1976). Radiosensitizers of hypoxic cells in solid tumours. *Cancer Treat. Rev.*, **3**, 227.

GOMER, C.J. & JOHNSON, R.J. (1979). Relationship between misonidazole toxicity and core temperature in C3H mice. *Radiat. Res.*, **78**, 329.

HALL, E.J. & ROIZIN-TOWLE, L. (1975). Hypoxic sensitizer; radiobiological studies at the cellular level. *Radiology*, **117**, 453.

HILL, S.A. & SIEMANN, D.W. (1984). Chemopotentiation *in vivo*: No loss of sensitization with fractionation. *Br. J. Cancer*, **50**, 509.

HIRST, D.G. & BROWN, J.M. (1982). The therapeutic potential of misonidazole enhancement of alkylating agent cytotoxicity. *Int. J. Radiat. Oncol. Biol. Phys.*, **8**, 639.

NCNALLY, N.J. (1982). Enhancement of chemotherapy agents. *Int. J. Radiat. Oncol. Biol. Phys.*, **8**, 593.

MCNALLY, N.J., HINCHLIFFE, M. & DE RONDE, J. (1983). Enhancement of the action of alkylating agents by single high, or chronic low doses of misonidazole. *Br. J. Cancer*, **48**, 271.

MILLAR, B.C. (1982). Hypoxic cell radiosensitizers as potential adjuvants to conventional chemotherapy for the treatment of cancer. *Biochem. Pharmacol.*, **31**, 2439.

MURRAY, J.C., RANDHAWA, V. & DENEKAMP, J. (1987). The effects of melphalan and misonidazole on the vasculature of a murine sarcoma. *Br. J. Cancer*, **55**, 233.

RANDHAWA, V.S., STEWART, F.A., DENEKAMP, J. & STRATFORD, M.R.L. (1985). Factors influencing the chemosensitization of melphalan by misonidazole. *Br. J. Cancer*, **51**, 219.

SAPIRSTEIN, L.A. (1958). Regional blood flow by fractional distribution of indicators. *Am. J. Physiol.*, **193**, 161.

SHELDON, P.W. & GIBSON, P. (1984). Effect of the nitroimidazole Ro-03-8799 on the activity of chemotherapeutic agents against a murine tumour *in vivo*. *Br. J. Cancer*, **49**, 291.

SIEMANN, D.W. (1984). Modification of chemotherapy by nitroimidazoles. *Int. J. Radiat. Oncol. Biol. Phys.*, **10**, 1595.

SMITH, K.A., HILL, S.A. & DENEKAMP, J. (1987). Hoechst 33342 as a vascular marker in tumours. *Br. J. Radiol.*, **60**, 102.

SONG, C.W. & LEVITT, S.H. (1970). Effect of irradiation on vascularity of normal tissues and experimental tumour. *Radiology*, **94**, 445.

STRATFORD, I.J. & ADAMS, G.E. (1977). The effect of hyperthermia on the differential cytotoxicity of the hypoxic cell radiosensitizer Ro-07-0582 on mammalian cells *in vivo*, *Br. J. Cancer*, **35**, 307.

TAMULEVICIUS, P., LUSCHER, G. & STREFFER, C. (1987). Effects on intermediary metabolism in mouse tissues by Ro-03-8799. *Br. J. Cancer*, **56**, 315.

TWENTYMAN, P.R. & WORKMAN, P. (1983). An investigation of the possibility of chemosensitization by clinically achievable concentrations of misonidazole. *Br. J. Cancer*, **47**, 187.